# Complications of cold *versus* hot snare polypectomy of 10–20 mm polyps: A retrospective cohort study

**DOI:** 10.1002/jgh3.12243

**Published:** 2019-08-18

**Authors:** Shara N Ket, Dileep Mangira, Allysia Ng, Douglas Tjandra, Ja H Koo, Richard La Nauze, Andrew Metz, Alan Moss, Gregor Brown

**Affiliations:** ^1^ Department of Gastroenterology Alfred Health Melbourne Victoria Australia; ^2^ Central Clinical School Monash University Melbourne Victoria Australia; ^3^ Department of Endoscopic Services Western Health Melbourne Victoria Australia; ^4^ Department of Medicine – Western Health, Melbourne Medical School University of Melbourne Melbourne Victoria Australia; ^5^ Department of Gastroenterology Melbourne Health Melbourne Victoria Australia; ^6^ Epworth Hospital Melbourne Victoria Australia

**Keywords:** adverse events, bleeding, cold snare, endoscopy, hot snare, polypectomy, sessile polyps

## Abstract

**Background and Aim:**

Cold snare polypectomy is safe and efficacious for removing polyps <10 mm with reduced rates of delayed postpolypectomy bleeding and postpolypectomy syndrome. This technique can also be used for sessile polyps ≥10 mm; however, further evidence is required to establish its safety. The aim of this study was to compare intraprocedure and postprocedure adverse events in patients who underwent cold (CSP) *versus* hot snare polypectomy (HSP) of 10–20 mm sessile colonic polyps.

**Methods:**

Electronic medical records and endoscopy reports of all patients who underwent polypectomy for Paris 0‐IIa, Is, or 0‐IIa + Is 10–20 mm colonic polyps between January 2015 and June 2017 at three tertiary academic hospitals and one private hospital were retrospectively reviewed. Data on patient demographics, polyp characteristics, method of polypectomy, and intraprocedural and postpolypectomy adverse events were collected.

**Results:**

A total of 408 patients (median age 67, 50% male) had 604 polyps, 10–20 mm in size, removed. Of these, 258 polyps were removed by HSP, with a median size of 15 mm (interquartile range [IQR] 12–20), compared to 346 polyps that were removed by CSP, with median size of 12 mm (IQR 10–15), *P* < 0.001. In the HSP group, 15 patients presented with postprocedure complications, including 11 with clinically significant bleeding, 2 with postpolypectomy syndrome, and 2 with abdominal pain. This compares with no postpolypectomy complications in the CSP group, *P* < 0.001.

**Conclusion:**

In this study, CSP was not associated with any postpolypectomy adverse events. CSP appears to be safer than HSP for removing 10–20 mm‐sized sessile polyps. A prospective multicenter study has been commenced to verify these findings and to assess the efficacy of CSP for the complete resection of polyps of this size.

## Graphical abstract

Cold snare polypectomy of 10–20 mm sessile polyps is safe, and no complications were seen when compared with hot snare.

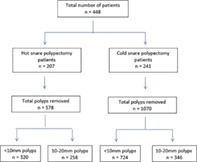

## Introduction

Hot snare polypectomy (HSP) has traditionally been the technique of choice for polypectomy and is performed using electrosurgical current delivered through a polypectomy snare as the polyp is transected. The rationale for this has been to minimize intraprocedural bleeding by cauterizing the transected tissue and provide additional transection power, and theoretically, the thermal injury could ablate any residual tissue at the polypectomy margin. Although the polypectomy site is often clean in appearance, and hemostasis is achieved immediately after HSP, there is a risk of postprocedure bleeding, perforation, and postpolypectomy syndrome. The rates of delayed postpolypectomy bleeding are estimated to range between 0.04 and 7.8%.^1^, ^2^, ^3^, ^4^, ^5^ The risk increases with increasing polyp size, right‐sided location, advanced histology, and use of antithrombotic agents.^4^, ^6^, ^7^, ^8^ Perforation is an uncommon but clinically significant event, occurring at a rate of 1.4–1.5%.^9^, ^10^

Cold snare polypectomy (CSP) is increasingly being used as the preferred method of polypectomy, particularly for small polyps.^11^ An increasing number of publications suggest that CSP is at least as safe as HSP, particularly for polyps <10 mm in size,^12^, ^13^, ^14^, ^15^ and efficacy data are trending in the same direction.^16^, ^17^, ^18^, ^19^ The aim of this study was to evaluate the intraprocedural and postprocedural adverse events following CSP *versus* HSP for sessile polyps 10–20 mm in size.

## Methods

### *Study design*

Electronic medical records and endoscopy reports of sequential patients who underwent colonoscopy between January 2015 and June 2017, at three academic tertiary hospitals and one private hospital, were retrospectively reviewed. Patients included in this study underwent HSP or CSP of Paris classification 0‐IIa, Is or 0‐IIa + Is polyps 10–20 mm in size based on the endoscopy report. The number of additional <10 mm polyps removed was also recorded. Exclusion criteria included any participant who had a polypectomy of a polyp >20 mm, if a combination of hot and cold snare techniques for 10–20 mm polyps were used, and if polyps <10 mm were removed by hot snare. Pedunculated polyps were not included as the possibility of a large vessel in the stalk feeding the polypoid head may increase the risk of bleeding after CSP, and this was not the focus of the present study.

This multicenter retrospective study was approved by the Alfred Health Ethics Committee, Melbourne, Australia.

### *Study population*

The data collected included patient age, gender, indications for colonoscopy, polyp location, size of polyps, polyp Paris classification, histological polyp characteristics, method of polypectomy, and intraprocedural and postprocedural adverse events within 14 days following colonoscopy. Intraprocedural adverse events that were studied included bleeding and resection of the muscularis propria, also known as “target sign” or perforation.^20^ Postprocedure adverse events included clinically significant bleeding that was defined as requiring admission to hospital or intervention, perforation, and postpolypectomy syndrome. Minor per‐rectal bleeding that did not require hospital admission or intervention was not included as a postprocedural adverse event. Requirements for the intraprocedural use of clips; soft coagulation via snare tip; or the need for postprocedural blood transfusion, repeat colonoscopy, angiography, or surgery were recorded.

### *Equipment*

Sedation was administered by an anesthetist using combinations of propofol, fentanyl, alfentanyl, and midazolam. Colonoscopic polypectomy was performed using colonoscopes (Olympus 190 series; Olympus Medical Systems Tokyo, Japan), cold snares (Exacto snare, with snare size of 9 mm; US endoscopy), and hot snares (Olympus SnareMaster snare, with snare size of 10, 15, or 20 mm). When submucosal injectate was used, it comprised a combination of Gelofusine and methylene blue, with or without adrenaline (1:100 000), as per proceduralist's preference. ERBE Endocut was used for HSP.

### *Statistical analysis*

Nonparametric data were presented as median and interquartile range, with statistical comparison among different groups performed using chi‐squared and Mann–Whitney U test. Multivariate logistic regression analysis was used to determine the effect of variables on delayed postpolypectomy adverse events. All statistical analyses were performed using SPSS Statistics, version 23 (SPSS Inc., IBM Corp. Armonk, NY, USA).

## Results

### *Participant characteristics*

A total of 448 patients were included; 50% were male. There was no statistically significant difference in the median age or number of patients on antithrombotic agents (Table 1).

**Table 1:** Participant characteristics

	Hot snare polypectomy (*n* = 207)	Cold snare polypectomy (*n* = 241)	*P* value
Median age (IQR)	69 (59–75)	67 (55–74)	NS
Male	57%	45%	0.008
Antithrombotic use total	25	24	NS
Antiplatelet (excluding aspirin)	9	4	
Warfarin	6	7	
Direct oral anticoagulants	10	11	
Therapeutic enoxaparin	0	2	
Median of total number of polypectomies per patient (range)	2 (1–4)	3 (2–6)	<0.001
Median number of polypectomies <10 mm per patient (IQR)	1 (0–2)	2 (1–4)	<0.001
Median number of polypectomies 10–20 mm per patient (IQR)	1 (1–1)	1 (1–2)	NS
Immediate complications	–	–	<0.001
Bleeding	15	3	
Clips	9	2	
Soft tip coagulation	6	1	
Target sign	1	0	
Delayed complications	15	0	<0.001
Bleeding	11	0	
Conservative management	5	0	
Patients requiring blood transfusion	4	0	
Further intervention	–	–	
Endoscopic	3	0	
Angiography	1	0	
Surgical	1	0	
Abdominal pain	2	0	
Postpolypectomy syndrome	2	0	
Perforation	0	0	

IQR, interquartile range; NS, no significance.

**Figure 1:**
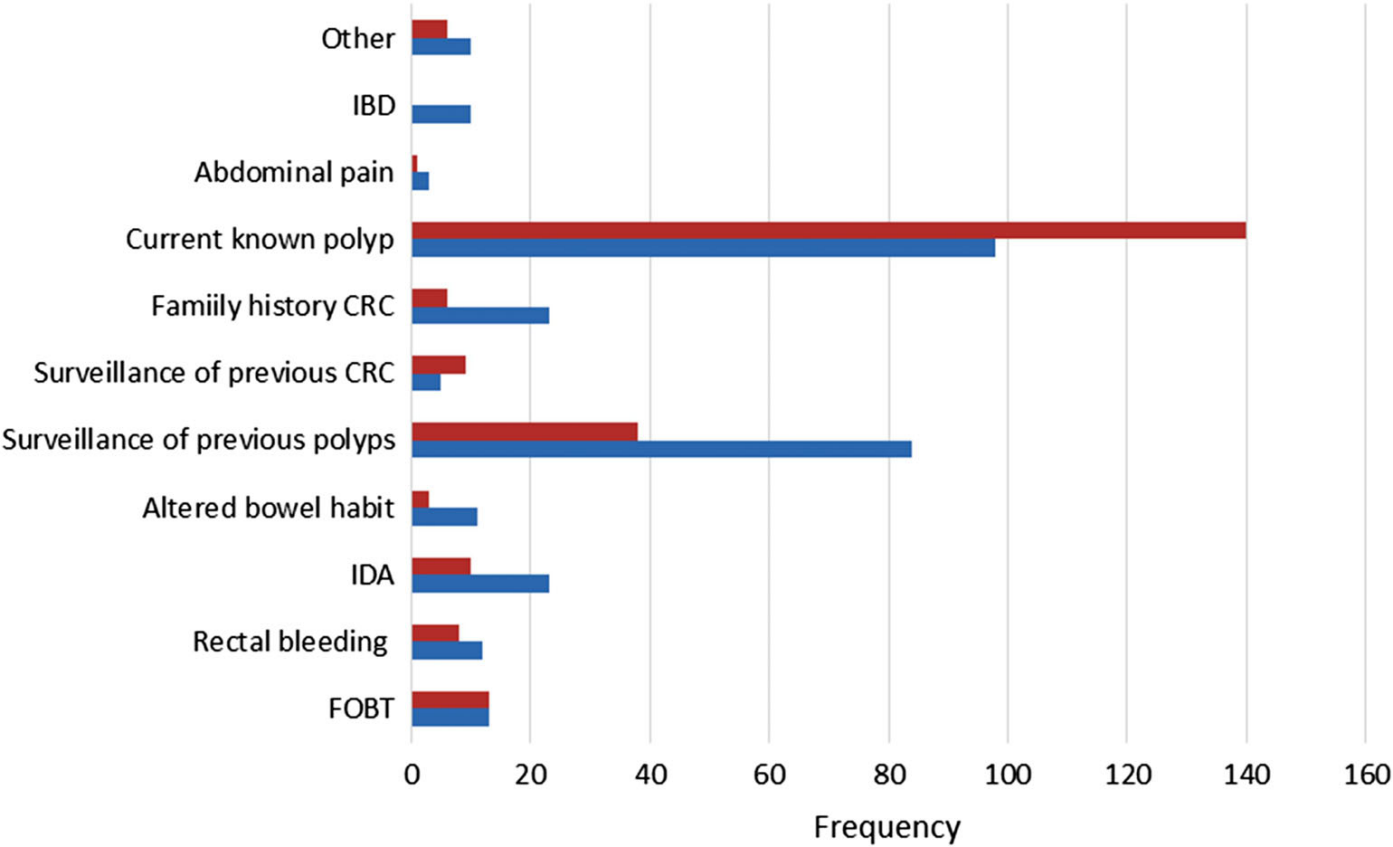
Indication for colonoscopy. (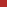), Hot snare polypectomy; (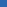), cold snare polypectomy. CRC, colorectal cancer; FOBT, faecal occult blood test; IBD, inflammatory bowel disease; IDA, iron deficiency anaemia; NS, no significance.

A total of 167 patients had two or more indications for colonoscopy. The most common indication for colonoscopy was therapy of a known polyp (*n* = 238), with 140 of 238 polyps removed by HSP. Polyp surveillance was the next most common indication for colonoscopy, with 84 of 122 undergoing CSP (Fig. 1).

### *Polyp characteristics*

**Figure 2:**
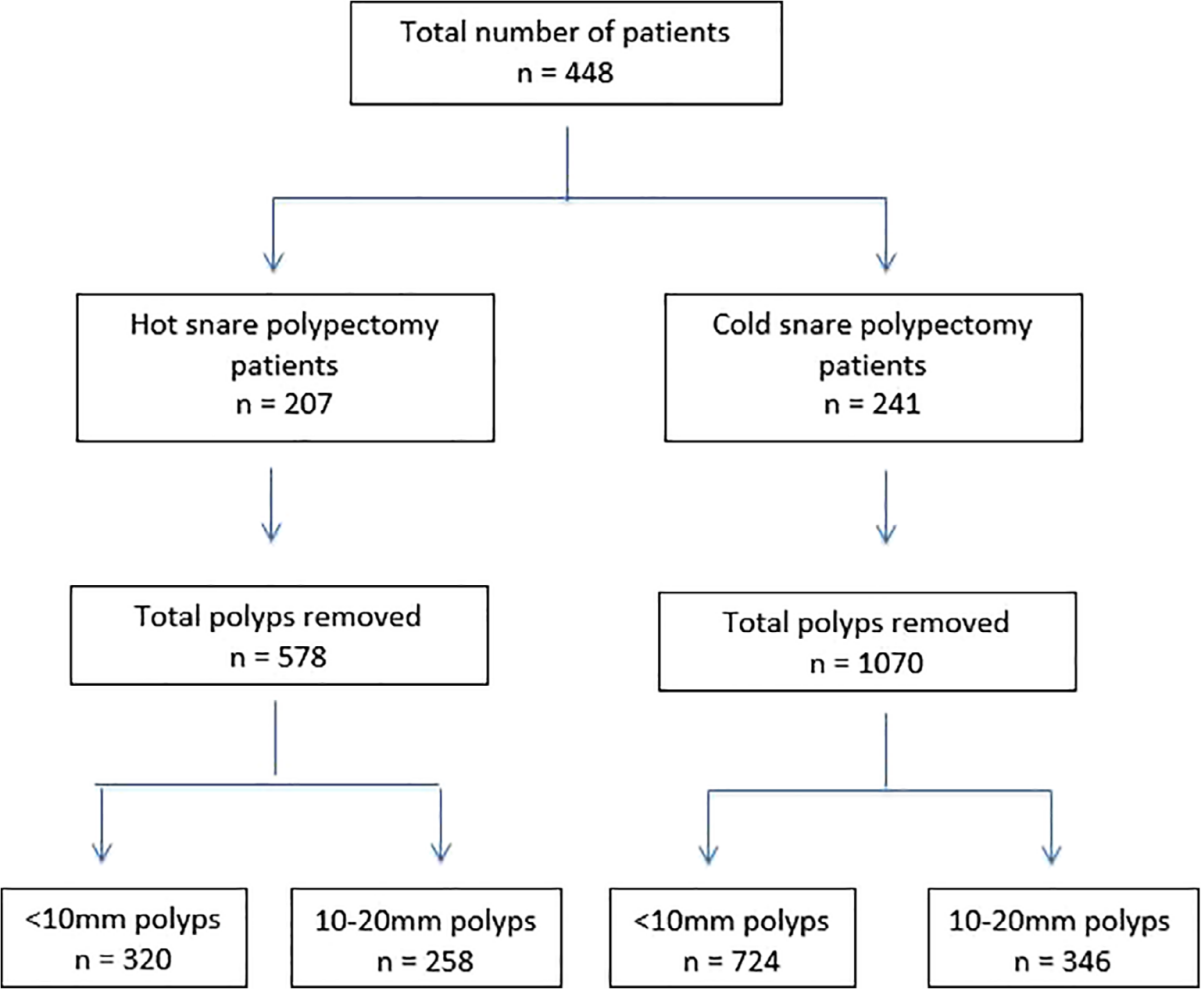
Flow chart of number of patients in each group, polyp sizes, and polypectomy method.

A total of 1649 polyps were removed; of these, 604 were polyps were 10–20 mm in size. The remaining 1044 were polyps <10 mm in size and removed by cold snare. Of the patients, 241 had 346 polyps 10–20 mm in size removed by CSP, and 207 patients had 258 polyps 10–20 mm in size removed by HSP (Fig. 2). The median polyp size was significantly larger in the hot than cold snare group (15 mm [interquartile range: IQR 12–20 mm] compared with 12 mm [IQR 10–15 mm], respectively; *P* = 0.001). However, the median number of total polypectomies per patient was significantly greater in the cold than hot snare group (3 [IQR 2–6] compared with 2 [IQR 1–4], respectively; *P* = 0.001). Sessile serrated polyps (54 *vs* 28%; *P* < 0.001) and hyperplastic polyps (5 *vs* 1%; *P* = 0.012) were more likely to be removed by cold snare and adenomas (37 *vs* 63%; *P* < 0.001) and cancer (0 *vs* 2%); *P* = 0.03) by hot snare. Submucosal injection to lift polyps was used more frequently for HSP (92%) compared with CSP (32%), *P* < 0.001. Of the 10–20 mm‐sized polyps removed by cold snare, 63% did not use a submucosal lift (Table 2).

**Table 2:** Polyp characteristics of 10–20 mm polyps

	Hot snare polypectomy (*n* = 258)	Cold snare polypectomy (*n* = 346)	*P* value
Median size of 10–20 mm polyps (IQR)	15 (12–20)]	12 (10–15)	<0.001
Location			0.03
Right colon	177 (69%)	266 (77%)	
Left colon	80 (31%)	80 (23%)	
Endoscopic appearance			
0‐IIa	159 (62%)	234 (68%)	NS
Is	81 (31%)	107 (31%)	NS
0‐IIa + Is	17 (7%)	1 (1%)	<0.001
Histology			
SSP	73 (28%)	187 (54%)	<0.001
Tubulo/tubulovillous adenoma	163 (63%)	127 (37%)	<0.001
With low‐grade dysplasia	145/163	125/127	0.002
With high‐grade dysplasia	18/163	2/127	0.02
Hyperplastic	4 (1%)	19 (5%)	0.012
Cancer	6 (2%)	0	0.03
Other	10 (4%)	13 (4%)	NS
Resection type			
En bloc	119 (46%)	105 (30%)	<0.001
Piecemeal	138 (53%)	241 (70%)	<0.001
Submucosal lift used	243 (94%)	128 (37%)	<0.001

IQR, interquartile range; SSP, sessile serrated polyp.

### *Adverse events*

There were 15 postprocedural adverse events that all occurred in patients who underwent HSP. These included 11 with clinically significant postprocedure bleeding, 2 with abdominal pain, and 2 with postpolypectomy syndrome. This compares with no postpolypectomy complications in the CSP group, *P* < 0.001. All 15 patients were admitted to hospital.

Of the patients with bleeding, five were conservatively managed and discharged after 1–2 days; two required blood transfusion only; one required blood transfusion and endoscopic intervention; one required endoscopic intervention only; and one required blood transfusion, endoscopic intervention, and angiographic embolization. Four patients were admitted with postprocedure abdominal pain; two of these patients fulfilled criteria for post‐polypectomy syndrome, but all were admitted for a maximum of 2 days.

One patient had an intraprocedural target sign, and despite the recognition of this and intraprocedural clipping, the patient had a major PR bleed 2 days postprocedure, requiring intensive care unit. Polyp histology demonstrated malignancy, so in the context of the major delayed bleed, a decision was made to perform an open right hemicolectomy.

Two patients who had postprocedural bleeding were on rivaroxaban. The first patient had four polyps removed; three were less than 1 cm in size, and the fourth was 18 mm in size, removed by hot snare. The rivaroxaban was withheld 4 days prior to the procedure and was recommenced 5 days postprocedure, and the patient presented with rectal bleeding 7 days postprocedure. The second patient has 17 polyps removed; 15 were less than 1 cm in size, and the remaining two were removed by hot snare and were 15 and 18 mm in size. This patient had rivaroxaban withheld 3 days prior to the procedure and was recommenced on it 2 days postprocedure, presenting with PR bleeding 3 days postprocedure. Both patients were conservatively managed without the need for blood transfusion and were discharged 2 days after presenting with postprocedure bleeding.

### *Other factors*

A multivariate analysis in patients who underwent HSP did not find that size, histology, submucosal lift, polyp location, or en bloc or piecemeal resection had any significant effect on postprocedure adverse events (Table 3).

**Table 3:** Multivariate logistic regression for delayed postpolypectomy bleeding for hot snare polypectomy

	Odds ratio (95% CI)	*P* value
Polyp size (10–15 *vs* 16–20 mm)	1.44 (0.37–5.67)	0.60
Location (right *vs* left colon)	1.19 (0.13–10.96)	0.80
Histology	1.00 (0.641–1.57)	0.98
Resection type (en bloc *vs* piecemeal)	0.89 (0.23–3.40)	0.87
Submucosal lift used (no *vs* yes)	1.19 (0.13–10.96)	0.88

CI, confidence interval.

## Discussion

Despite the increasing pool of published literature on CSP, polypectomy strategies among gastroenterologists remain highly variable. With regard to safety, HSP results in eschar formation with the risk of postprocedure adverse events, such as postpolypectomy bleeding, postpolypectomy syndrome, or perforation. These risks are significantly mitigated by using CSP.

This study found that all postprocedure adverse events requiring admission, blood transfusion, or intervention occurred in patients who had HSP. In comparison, no postprocedure complications were seen in the CSP group (*P* < 0.001). Despite the hot snare group having a larger median polyp size than the cold snare group, the total number of polyps removed was significantly greater in the cold snare group.

Published literature supports the safety of CSP for the removal of small polyps. A retrospective review from Japan looked at the feasibility of CSP of subcentimeter polyps. In that study, 234 polyps were removed from 61 patients, with 3.4% experiencing intraprocedural adverse events that included eight lesions requiring endoscopic clipping alone. Three patients had delayed adverse events of minor postprocedural bleeding.^1^ A prospective randomized study^12^ in 2011 compared HSP *versus* CSP for 3–8 mm‐sized polyps. A total of 414 patients were recruited, with 543 polyps removed by cold snare and 540 removed by hot snare. A greater number of patients who underwent CSP had intraprocedural bleeding (*n* = 19 compared to *n* = 2, respectively); however, this bleeding resolved spontaneously with no intervention being required. Neither group experienced postprocedure bleeding. While, previously, endoscopists may have been concerned about intraprocedural bleeding following CSP, in our experience, intraprocedural bleeding following CSP almost always ceases spontaneously, and observation for a short time almost always obviates the need for intervention. In the rare case where bleeding persists, endoscopic clipping is an effective management option.

The risk of delayed postpolypectomy bleeding increases with polyp size.^4^, ^7^ However, two studies have looked at CSP of >10 mm polyps, and as size increases beyond 10 mm, CSP appears to be at least as safe as HSP and without a notable increase in intraprocedural or postprocedural bleeding.^14^, ^21^

Antithrombotic agents pose a concern for endoscopists due to the possible increased risk of intraprocedural and postprocedural bleeding following polypectomy. However, bleeding risk must be balanced against the cardiovascular risk to the patient due to the temporary interruption of antithrombotic agents. In this study, there was no statistical difference in the number of patients on antithrombotic agents who had CSP *versus* HSP. In the present study, two patients who were on direct‐acting anticoagulants had postprocedure bleeding complications; however, these patients had appropriate periendoscopic interruption of these agents.^5^ Two studies have specifically looked at CSP on antithrombotics.^22^, ^23^ Overall, the risk of delayed postpolypectomy bleeding following CSP was acceptably low, with only 2 of 207 patients on therapeutic antithrombotic agents experiencing delayed postpolypectomy bleeding. Both of these patients were on direct oral anticoagulants (dabigatran and apixaban).^22^, ^23^

Complete resection is now considered a powerful indicator of the quality of colonoscopy.^24^ HSP has conventionally been used, which is thought to provide thermal ablation of polyp at the polypectomy margin. However, the prospective, multicenter CARE study demonstrated that 10.1% of 346 neoplastic polyps removed by hot snare were incompletely resected.^25^ A retrospective study looking at vertical and horizontal histopathological margins in CSP aimed to characterize complete resection rates. They found that 25.1% of margins were unclear, while 70.5% were negative and 4.4% were positive.^26^ In contrast, a prospective observational study assessed the margins of CSP by performing a submucosal injection into the polypectomy site and then performed an en bloc hot snare resection of the entire CSP margin; this study found a 3.9% incomplete resection rate.^17^ It has been suggested that incomplete resection during CSP can be minimized by ensuring there is a 2 mm margin of normal mucosa around the polyp prior to polypectomy.^27^ However, this is limited to small polyps if the resection is en bloc. Further evidence is required to determine the efficacy of CSP, particularly piecemeal CSP, compared with HSP for polyps in the size range of 10–20 mm, where en bloc resection may not be possible. The safety inherent in CSP potentially allows for the aggressive resection of the polyp and margins to ensure complete resection, but this requires a prospective, multicenter study, which is currently underway.

The major limitation of our study is its retrospective nature. This includes potential selection bias in the endoscopists' choice of HSP *versus* CSP for particular polyps, unrecognized patient factors, or other undocumented factors related to the polypectomy that cannot be determined from a retrospective review. If a patient presented to another hospital with a postprocedural adverse event, this would not have been captured. However, the likelihood of this is low as patients were instructed to present to the hospital where the procedure was performed in the event of any postprocedure concerns. The number of documented intraprocedural bleeding adverse events was low, particularly in the cold snare group; however, intraprocedural bleeding was only documented if endoscopic hemostatic techniques were required, and thus, any minor bleeding that was not clinically significant was not captured. However, this is arguably not a true limitation as such minor bleeding should not be considered of importance. The multivariate analysis comparing the effect of polyp characteristics on delayed adverse events was not significant, and this is conflicting with previous studies. It is likely that this relates to the small number of events and sample size, resulting in insufficient power to elicit a difference. Finally, we emphasize that this study did not assess the efficacy of CSP *versus* HSP for completeness of resection, but rather, it assessed the safety aspects of polypectomy only.

In conclusion, CSP appears safe for the removal of 10–20 mm sessile colonic polyps. All delayed adverse events were seen in patients who underwent HSP. There were no postprocedural adverse events in the CSP group, and this difference was statistically significant. Therefore, CSP appears safer than HSP for the removal of sessile 10–20 mm‐sized polyps. However, a prospective study of the efficacy of CSP for achieving complete polyp resection for polyps in this size range is required before recommendations can be made regarding the optimal polypectomy technique. A prospective multicenter study has been commenced to assess both the safety and efficacy of CSP for the resection of 10–20 mm‐sized sessile polyps.
